# The general movements assessment in term and late-preterm infants diagnosed with neonatal encephalopathy, as a predictive tool of cerebral palsy by 2 years of age: a scoping review protocol

**DOI:** 10.1186/s13643-020-01358-x

**Published:** 2020-07-04

**Authors:** Judy Seesahai, Maureen Luther, Carmen Cindy Rhoden, Paige Terrien Church, Elizabeth Asztalos, Rudaina Banihani

**Affiliations:** 1 Sunnybrook Health Sciences Centre, Toronto, Canada; 2 University of Toronto, Toronto, Ontario Canada

**Keywords:** Neonatal encephalopathy, General movement assessment, Prechtl, Hypoxia-ischemia encephalopathy, Cerebral palsy, Infants/neonates, Term babies, Preterm babies, Motor development

## Abstract

**Background:**

Prediction of long-term neurodevelopmental outcomes remains an elusive goal for neonatology. Clinical and socioeconomic markers have not proven to be adequately reliable. The limitation in prognostication includes those term and late-preterm infants born with neonatal encephalopathy. The General Movements Assessment tool by Prechtl has demonstrated reliability for identifying infants at risk for neuromotor impairment. This tool is non-invasive and cost-effective. The purpose of this study is to identify the published literature on how this tool applies to the prediction of cerebral palsy in term and late-preterm infants diagnosed with neonatal encephalopathy and so detect the research gaps.

**Methods:**

We will conduct a systematic scoping review for data on sensitivity, specificity, positive, and negative predictive value and describe the strengths and limitations of the results. This review will consider studies that included infants more than or equal to 34 + 0 weeks gestational age, diagnosed with neonatal encephalopathy, with a General Movements Assessment done between birth to six months of life and an assessment for cerebral palsy by at least 2 years of age. Experimental and quasi-experimental study designs including randomized controlled trials, non-randomized controlled trials, before and after studies, interrupted time-series studies and systematic reviews will be considered. Case reports, case series, case control, and cross-sectional studies will be included. Text, opinion papers, and animal studies will not be considered for inclusion in this scoping review as this is a highly specific and medical topic. Studies in the English language only will be considered. Studies published from at least 1970 will be included as this is around the time when the General Movements Assessment was first introduced in neonatology as a potential predictor of neuromotor outcomes. We will search five databases (MEDLINE, Embase, PsychINFO, Scopus, and CINAHL). Two reviewers will conduct all screening and data extraction independently. The articles will be categorized according to key findings and a critical appraisal performed.

**Discussion:**

The results of this review will guide future research to improve early identification and timely intervention in infants with neonatal encephalopathy at risk of neuromotor impairment.

**Systematic review registration:**

Title registration with Joanna Briggs Institute https://joannabriggs.org/ebp/systematic_review_register.

## Background

Prediction of long-term neurodevelopmental outcomes remains an elusive goal for neonatology. Clinical and socioeconomic outcome markers have not proven to be adequately reliable [1, 2]. The limitation in prognostication includes those term and late-preterm infants born with neonatal encephalopathy (NE).

NE describes those infants born with an atypical neurological exam and is by definition heterogeneous in etiology [3]. The specific etiology may not be clear for months to years later but the presentation is characterized by central nervous system disruption [4] and is associated with an increased risk for long-term neurodevelopmental challenges including cerebral palsy (CP). Infants presenting with NE are managed now with therapeutic hypothermia as the standard of care; this is presumptive management, and is time sensitive should the etiology be hypoxia/ischemia (hypoxic-ischemic encephalopathy (HIE)), in term and late-preterm infants [4, 5]. Therapeutic hypothermia reduces the likelihood of challenging outcomes by containing any potential ongoing neurological injury. It does not, however, completely eradicate the possibility of long-term neurodevelopmental disability [6].

For parents of infants affected by NE, the desire for accurate prognostication is of tantamount importance [7]. This information can guide decisions around early intervention and, in severe cases, withdrawal of care for those infants with severe involvement. For those infants that survive NE and are at increased risk for CP, recent international recommendations now call for early detection and intervention of CP in order to improve functional outcomes [1, 8, 9]. These recommendations are based on mounting evidence for better detection tools as well as the benefits of early intervention.

Historically, clinical and radiological predictors of neurological outcomes were used to classify the degree of NE. Severity scoring systems include the classical grading by Sarnat and Sarnat [10] in 1976, to the newer scores by Miller et al. [11] in 2004, with added parameters such as oral feeding difficulties and the presence of seizures. Radiologically, specific findings of diffusion restriction on magnetic resonance imaging (MRI) have been linked to later development of CP [4]. These predictors, however, were not sufficiently accurate [1, 2] and the high costs of imaging as well as shortages in access further restricts the utility. Neurological examinations have historically been limited in predictive value but recent emerging evidence with an observational tool, the General Movements Assessment (GMA) developed by Dr. Heinz Prechtl has demonstrated strong predictive value [12, 13].

The GMA is a non-invasive, cost-effective tool with demonstrated reliability for identifying infants at risk for neuromotor impairment [14]. General movements (GMs) are complex, highly variable, whole-body movements which emerge in the fetus and progress through an age-specific developmental trajectory, dissipating by the end of the first 4 to 5 months of life [13]. Developmental progression and variety, or lack thereof, are indicators of nervous system integrity and can reflect neurodevelopmental outcomes [15]. Cramped synchronized (CS) and absent fidgety movements are considered abnormal GMAs, demonstrating developmental stereotypy [13].

Several researchers have looked at the GMA from different aspects. A preliminary search of PROSPERO, MEDLINE, the Cochrane Database of Systematic Reviews, and the Joanna Briggs Institute (JBI) Database of Systematic Reviews and Implementation Reports was conducted to assess this research. There were two current systematic reviews on GMA, one in 2018 [16] and the other in 2017 [8]. In addition, eight older reviews were identified: seven systematic reviews [13, 17–22] and one literature review [23] done between 2001 and 2013. The search also revealed three pending reviews identified around the topic of the predictive value of GMA [24–26]. These pending reviews were all systematic reviews.

The key characteristics and main findings of the above reviews on GMA are presented in Table 1 in Appendix 1. In general, the latest systematic review, by Kwong et al. in 2018 [16], compared assessments of GMA and found that the Prechtl method had the best prediction of CP. In the 2017 systematic review by Novak et al. [9], their group reviewed the evidence for the best tools for early, accurate diagnosis and intervention in infants at risk for CP. They considered all gestational ages (GA) and all diagnoses for infants that were high-risk. They recommended a combined approach for early CP diagnosis including history, neuroimaging, standardized neurological, and standardized motor assessments, to facilitate timely diagnosis and intervention. The other systematic reviews and literature review were all more than 5 years ago with the latest in 2013 [13]. The findings of these older reviews are also summarized in Table 1 in Appendix 1. Similar to the latest two reviews, the older reviews either looked at preterms or all GA groups and diagnoses.

Of the three pending systematic reviews identified in PROSPERO, the oldest review protocol (Kwong et al.) [26] was registered in 2016 by similar authors of the 2018 review mentioned above. The next review protocol was registered in February 2018 by Raghuram et al. [24], and plans restrictions to preterms with all diagnoses, specifically examining automated movement recognition technology with the GMA. The third review protocol, registered in April 2018, by Angélica Valencia [25] is limited to preterm infants and is evaluating the type of method used for the recognition of the GMA, not the relationship of the GMA to neuromotor outcomes. None of these reviews specifically look at the population we identified for this scoping review, that is, term and late-preterm infants with NE. Thus, a gap exists in the literature to clearly identify the evidence for this specific population.

The objective of this review is therefore, to identify the scope of the research with regard to the GMA and its ability to predict CP, in term and late-preterm infants with a diagnosis of NE, and to identify the gaps in the literature.

## Methods/design

### Review question

The primary research question for this review is: What is the published data on the predictive value of the GMA for the diagnosis of CP by 2 years of age in infants born at term or late-preterm presenting with NE?

The secondary research question is: What is the gap in the literature when the GMA is used to predict CP by 2 years of age in infants born at term or late-preterm presenting with NE?

### Study design

A scoping method is chosen for this type of review as to fulfilling of the objective of the review it requires searching and assessing a wide range of research methodologies involving the use of the GMA in CP prediction. A scoping review will capture all types of relevant research on the topic in a systematic, transparent, rigorous, and reproducible manner. This scoping review will be conducted in accordance with the JBI methodology for scoping reviews [27]. The objectives, inclusion criteria, and methods for this scoping review are detailed in advance and documented in a proposal (included as Additional file 1). The title of our review was registered with JBI.

Inherent in the nature of the scoping review is the inclusiveness of a wide range of literature, and so we anticipate differences in the data quality. Critical appraisal and data synthesis therefore will be challenging in terms of conclusive evidence as opposed to in a systematic review. The scoping review methodology is however especially advantageous to our question as these types of reviews target areas that have not been comprehensively assessed before.

### Eligibility criteria

The participant, concept, context (PCC) framework for scoping reviews will be used to define the review focus and can be found in Table 2 in Appendix 2.

#### Participants

This review will consider studies that include infants ≥ 34 + 0 weeks GA diagnosed with NE with a GMA done between birth to 6 months of life and an assessment for CP by at least 2 years of age (Table 2 in Appendix 2).

Reviews with infants born with life threatening congenital abnormalities, congenital viral infections, an abnormal karyotype and metabolic disorders will be excluded. Those studies without a GMA or with any automated application of the GMA will also be excluded.

#### Concept

GMA as a predictor of CP by 2 years of age is the main concept. Studies that report on sensitivity, specificity, positive predictive value (PPV), and negative predictive value (NPV) will be considered for inclusion. Detailed definition of concepts can be found in Table 3 in Appendix 3.

#### Context

This review will consider studies that reported on infants with an existing diagnosis of NE managed in hospitals and diagnosed by the standard of care assessment of a neurological history and examination. Studies will be considered from all countries that have outcomes reported in the acute neonatal and in the follow-up period by 2 years of age. Studies in the English language only will be considered as there is no team member with adequate language skills to translate from any other language.

### Search strategy

A range of electronic databases will be searched to include medicine, nursing, allied health professions, sociology, psychology, education, and social work. This scoping review will consider both experimental and quasi-experimental study designs including randomized controlled trials, non-randomized controlled trials, before and after studies and interrupted time-series studies. Case reports, case series, case control, and cross-sectional studies will be included. In addition, systematic reviews that meet the inclusion criteria will be considered. Text and opinion papers will not be considered for inclusion in this scoping review as this is a highly specific and medical topic. Animal studies will not be included. Studies published from at least 1970 will be included as this is around the time when the GMA was first introduced in neonatology as a potential predictor of neuromotor outcomes [12]. The reference lists of articles will be scanned and experts in the infant developmental field will be consulted to identify studies relevant to our topic.

The search strategy will be phased, firstly created in Ovid MEDLINE using a combination of index terms and keywords around general movements, Prechtl, brain disease, HIE, and perinatal asphyxia. An initial limited search of Ovid MEDLINE, Embase, and PsychINFO was undertaken to identify articles on the topic (see Additional file 2). There were no previous similar reviews. The text words contained in the titles and abstracts of relevant articles, and the index terms used to describe the articles from this limited search will then be used to develop a more refined full search strategy in the second phase, for MEDLINE, Embase, PsychINFO, Scopus, and CINAHL (Appendix 3). The search strategy, including all identified keywords and index terms, will be adapted for each included information source.

### Study selection

EndNote X9 will be used for citation collation. Duplicates will be removed manually. Covidence will be used for screening by two independent reviewers (JS and ML). Disagreements will be resolved through a third reviewer (RB). The results of the search will be reported in a Preferred Reporting Items for Systematic Reviews and Meta-analyses extension for scoping reviews (PRISMA-ScR) flow diagram [28].

### Data extraction, analysis, and synthesis

Publications meeting the inclusion criteria will have a full text review to validate their eligibility. Each article will be assessed independently by two authors (JS and RB). Extraction will be done after full text screening using a data extraction tool developed by the reviewers. Excluded studies closely meeting the inclusion criteria will be included in a separate table as they may contain many elements of our inclusion criteria but not present separately the specific criteria of our interest. Further investigation of their data may provide significant results. Authors will be contacted to access further information and reassess eligibility of these studies. Excluded studies will be documented with reasons for their exclusion.

The data extracted from the identified studies will include specific details about the population, concept, and context. Two tables will be generated with the first table having information on the key characteristics of each study, including author, year of publication, geographical setting, type of study, demographics of the participants, period over which the study was conducted, the method of identification of neonates at high-risk, if therapeutic hypothermia was instituted as management for NE, type of spontaneous movement assessment used, age at which participants were assessed, the age at which CP was diagnosed, and the methods used for neurological examination in the studies. The second table will have information on the key findings, the predictive indices used for the GMA in relation to CP (sensitivity, specificity, PPV, and NPV), limitations of the studies, and where relevant, reasons for exclusion in the studies that met most but not all of the inclusion criteria. These lists will be iterative. As the process evolves, the data extraction form may require modification to ensure all relevant information is included. Additionally, even though this was a scoping review and does not require a critical appraisal, the critical appraisal tool for JBI [29] will help to identify differences and similarities between the included studies. The answers to the JBI critical appraisal tool will be detailed in a table.

## Discussion

The extracted data will be presented in diagrammatic or tabular form in a manner that aligns with the objective of this scoping review. A narrative summary will accompany the tabulated and/or charted results and will describe how the results relate to the reviews objective and question. The critical appraisal result will also be tabulated and this will be used to further identify the strengths and limitations of the studies as well as the key findings in relationship to the objective of this scoping review. The strengths and limitations of our scoping review method on the credibility of the results will be detailed. The discussion and conclusions will reflect on the implications for future research and patient management.

### Protocol amendments

Important amendments to the protocol will be reported with the results of the review.

#### What this study will add

This study will examine the scope of the literature with respect to the use of the GMA in NE for the prediction of CP. Assessment of the extent of the knowledge on this topic seems to have not previously been done. By inclusion of a critical appraisal of the available relevant literature, it will facilitate an appreciation of the quality of the existing knowledge in this area. It will therefore identify gaps in the research especially in the setting of NE management with therapeutic hypothermia.

### Supplementary information

**Additional file 1.** The General Movements Assessment in Term and Late-preterm newborns diagnosed with Neonatal Encephalopathy, as a predictive tool of Cerebral Palsy at two years of age - A Scoping Review

**Additional file 2.** Ovid MEDLINE search. Search conducted on Ovid MEDLINE(R), Ovid MEDLINE(R) Daily and Epub Ahead of Print, In-Process & Other Non-Indexed Citations 1946 to Present

## Figures and Tables

**Table 1:**
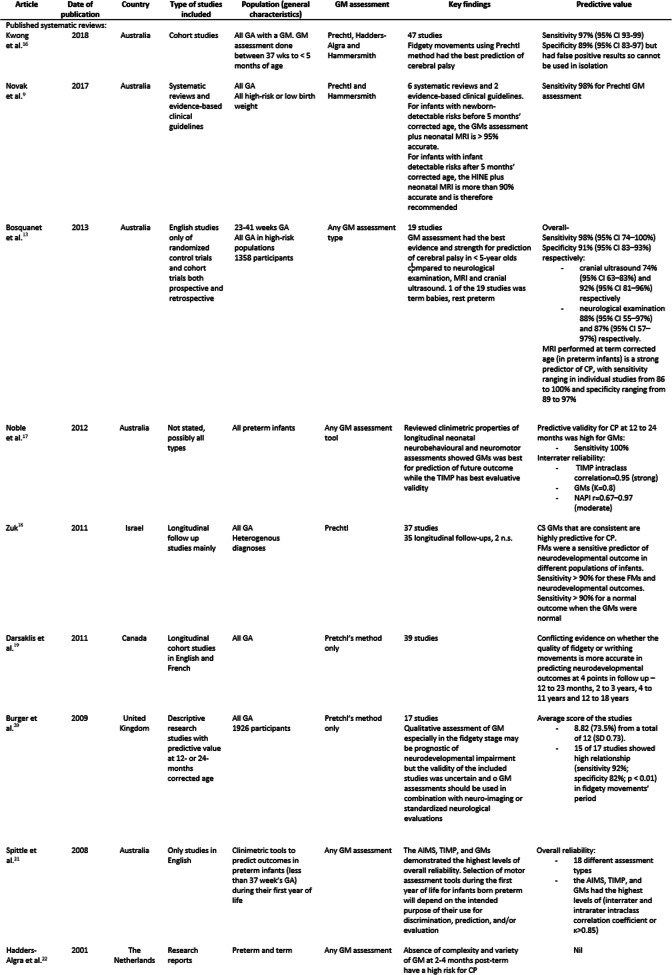

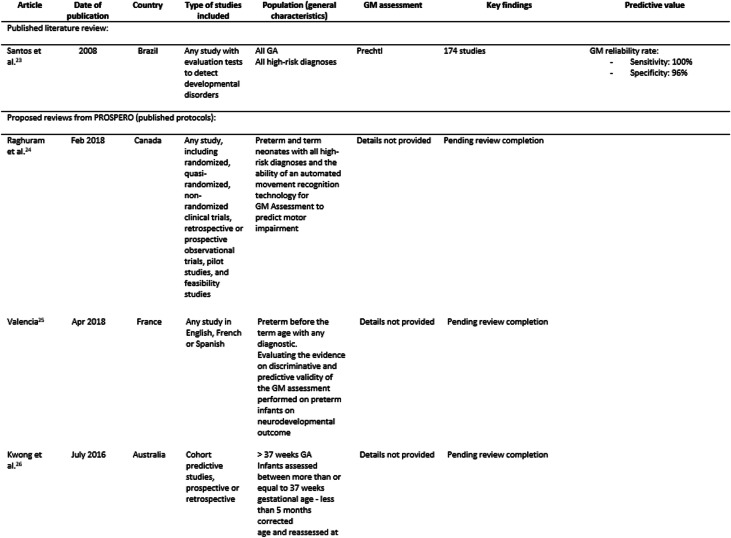
Summary of reviews (published and pending) on the general movements assessment and its predictive value for neuromotor outcomes

Note. AIMS=The Alberta infant motor scale, CI=confidence interval, CP=cerebral palsy, CS=cramped synchronized, GM=general movement, HINE=Hammersmith Infant Neurological Examination, NAPI=Neurobehavioural Assessment of the Preterm Infant, n.s. = not stated, TIMP=Test of infant motor performance, wks = weeks

## Data Availability

Data sharing is not applicable to this article as no datasets were generated or analyzed during the current study. Materials during the current study are available from the corresponding author on reasonable request.

## Appendix 2

**Table 2:** Inclusion and exclusion criteria for the prediction of CP by the GMA in late-preterm and term infants with NE

	Inclusion criteria	Exclusion criteria
Participants	Infants ≥ 34 + 0 weeks GA Diagnosis of NE GMA done between birth up to 6 months of life Assessment for CP by at least 2 years of age	Infants born with: - Life threatening congenital abnormalities - Congenital viral infections - An abnormal karyotype and - Metabolic disorders
Concept	GMA as a predictor of CP by 2 years of age is the main concept.	
Context	Studies that reported on the following: -Infants with NE managed in hospitals and diagnosed by the standard of care (neurological history and examination) -Studies from all countries that have outcomes reported in the acute neonatal and in the follow-up period by 2 years of age -Studies in the English language only	

Note. CP cerebral palsy, GA gestational age, GMA general movements assessment, NE neonatal encephalopathy

## Appendix 3

**Table 3:** Definitions of concepts

Concepts	Definition
Neonatal encephalopathy	A clinically defined syndrome of disturbed neurologic function in the earliest days of life in an infant born at or beyond 35 weeks of gestation, manifested by a subnormal level of consciousness or seizures, and often accompanied by difficulty with initiating and maintaining respiration and depression of tone and reflexes [3]
Late-preterm	Neonates ≥ 34 + 0 to 36 + 6 weeks GA [30]
Term	Neonates 37 + 0 to 42 + 6 weeks GA [30]
Cerebral palsy	A group of permanent disorders of the development of movement and posture causing activity limitations that are attributed to non-progressive disturbances that occurred in the developing fetal or infant brain [31]
General movements	These are spontaneous movements present from early fetal life until about 6 months of life. GMs are variable, complex movements that occur frequently, lasting long enough to be observed. The whole body is involved in a variable sequence of limbs, neck, and trunk movements. Waxing and waning in intensity, force, and speed, they have a gradual beginning and end. They involve rotations along the limb axis. Slight changes in direction are responsible for their fluid elegance. Impairment of the nervous system causes the loss of GMs complexity and variability resulting in monotonous and poor-quality movements. Specific abnormal GM patterns have been identified that reliably predict later cerebral palsy: (1) Cramped-synchronized GMs—a persistence of rigid movements that lack the normal fluidity. Contractions and relaxations occur almost concurrently in limb and trunk muscles. (2) The absence of fidgety GMs—fidgety movements are small movements of moderate speed with variable acceleration of neck, trunk, and limbs in all directions. Normally, they are the predominant movement pattern in an awake infant at 3 to 5 months [32].
General movements assessment	A comfortably dressed infant, preferably with bare arms and legs, is videoed in supine position. The duration of the video recording will depend on the age of the infant with premature infants requiring up to 30 to 60 minutes. Term age and older require 5 to 10 min of optimal recording. This recording does not require the observer’s presence. The trained observer reviews the recording later. The assessment is based on global visual Gestalt perception without acoustic signal to reduce distraction. Two to three recordings of the preterm, one recording at term or early post-term age or both, and at least one recording between 9- and 15-week post-term forms the basis of a developmental trajectory. An individual developmental trajectory indicates the consistency or inconsistency of normal or abnormal findings [32].
Sensitivity	The proportion of true positives that are correctly identified in a sample, or the true positive rate [33].
Specificity	The proportion of true negatives that are correctly identified in a sample, or the true negative rate [33].
Positive predictive value	The proportion of patients with positive test results who are correctly diagnosed [34].
Negative predictive value	The proportion of patients with negative test results who are correctly diagnosed [34].

*Note. GA* gestational age, *GMs* general movements

## Abbreviations

CP: Cerebral palsy
CS: Cramped synchronized
GMs: General movements
GA: Gestational age
GMA: General Movements Assessment
HIE: Hypoxic-ischemic encephalopathy
JBI: Joanna Briggs Institute
MRI: Magnetic resonance imaging
NE: Neonatal encephalopathy
NPV: Negative predictive value
PCC: Participant, concept, context
PPV: Positive predictive value
PRISMA-ScR: Preferred Reporting Items for Systematic Reviews and Meta-analyses extension for scoping review
