# Supplementary material for: The general movements assessment in term and late-preterm infants diagnosed with neonatal encephalopathy, as a predictive tool of cerebral palsy by 2 years of age: a scoping review protocol
Source: Syst Rev. 2020 Jul 4;9:154. doi: 10.1186/s13643-020-01358-x (PMC7335433; doi:10.1186/s13643-020-01358-x)
Supplement: Supplementary file 1 — Additional file 1. The General Movements Assessment in Term and Late-preterm newborns diagnosed with Neonatal Encephalopathy, as a predictive tool of Cerebral Palsy at two years of age - A Scoping Review [file 13643_2020_1358_MOESM1_ESM.docx]

The General Movements Assessment in Term and Late-preterm newborns diagnosed with Neonatal Encephalopathy, as a predictive tool of Cerebral Palsy at two years of age - A Scoping Review.

# Authors

Judy Seesahai, Maureen Luther, Carmen Rhoden, Paige Church, Elizabeth Azstalos, Rudaina Banihani - Sunnybrook Health Sciences Centre, Toronto, Canada

# Introduction

Better outcomes are the expectation of the quality improvement (QI) movement that is currently taking the healthcare sector by storm. Effectiveness, efficiency and patient-centeredness is at the core of QI culture and has been adopted by Health Quality Ontario^1^. Unfortunately, predictors of outcomes have, as in many cases in medicine, not been as reliable as either the medical providers or their patients have hoped. In the perinatal world this truth is no different. The potential insults on the developing brain are multi-factorial and run throughout pregnancy and early newborn life. Having escaped the prematurity issues for the most part, the late-preterm and term neonatal brains are still significantly prone to the impacts of perinatal insults and may manifest this at birth as neonatal encephalopathy.

Neonatal encephalopathy (NE) is defined as a disorder of heterogeneous etiology that results in disruption of the central nervous system^2^. Neonates with encephalopathy are at risk for long-term neurodevelopmental challenges. In the intrapartum period hypoxia/ischemia is thought to be one of the causes of NE. For newborns with moderate to severe neonatal encephalopathy, caused presumably by hypoxia/ischemia (Hypoxic-Ischemic encephalopathy or HIE), therapeutic hypothermia is now the standard of care in many experienced centers based on controlled trials and meta-analyses showing improvement of outcomes with this intervention^2,3^. Some institutions do not have the resources for this intervention to be the standard of care. Furthermore, even in those countries with the ability to provide this treatment option, the risk of challenging outcomes later in life is not entirely eliminated. These neonates with NE are usually monitored clinically from birth, for evidence of neurological issues both in the short and long term. Already anxious parents of neonates who have suffered from HIE, look forward to as accurate as possible information from medical experts concerning the potential outcomes for their children^4^. Maybe, more importantly to both parents and medical providers, early identification of neonates at risk, serves to guide appropriate therapy or even in severe cases assist with decision making for withdrawal of care. In many cases early detection of cerebral palsy coupled with early intervention has been shown to improve outcomes^5^.

Both clinical and radiological predictors of neurological outcomes have been used, including looking at impairments in the neurological examination such as the presence and extent of seizures, inability to orally feed, findings of specific signs such as diffusion restriction on neonatal magnetic resonance imaging (MRI)^2^. These predictors have not always been found to be very accurate^6^. In addition to lack of accurate predictive values, MRI imaging is costly and access to this modality may be restricted in settings with limited human and mechanical infrastructure.

The General Movements (GM) Assessment is a clinical assessment tool which looks at the loss of primitive functions and the acquisition of neurodevelopmentally more mature skills in neonates and infants^7^. The progression of development, or lack thereof is a reflection of the integrity of the nervous system and has been found to be associated with better or worse neurodevelopmental outcomes respectively^8^.

A preliminary search of PROSPERO, MEDLINE, the Cochrane Database of Systematic Reviews and the JBI Database of Systematic Reviews and Implementation Reports was conducted. There was one current systematic review in 2018^9^ and five older reviews from 2001 to 2013^6,10,11,12,7^. Additionally, three systematic reviews were identified that were underway around the topic of the predictive value of GM Assessments. The current systematic review in 2018 was by Kwong et al.^9^. In that review the authors looked at the predictive validity for spontaneous early infant movement for cerebral palsy in later life with three assessments namely: Prechtl, Hadders-Algra and the Hammersmith assessments. They found that of the three, the Prechtl method had the best prediction of cerebral palsy. The study population here was all comers with a GM Assessment done between 37 weeks and less than 5 months of age. They did not specifically look at the population we identified in this systematic review, that is, late-preterm and term. They also did not distinguish the predictive value for the high-risk population of NE in this age group. Thus, their study aim was different from our study. The other systematic reviews were all more than 5 years ago with the latest in 2013 ^6^. There were three pending systematic reviews identified in PROSPERO. The oldest was registered in 2016 by similar authors of the 2018 review mentioned above^13^. In the proposed review the population is limited to full term neonates only (more than 37 weeks gestational age). Additionally, again, they are not looking specifically at those neonates diagnosed with NE. The second protocol was registered in February 2018 by Raghuram et al., and plans to look only at preterm neonates with all diagnoses, not term^14^. They intend to look at the GM Assessment using automated movement recognition technology and they did not state an intention to divide into a subgroup with NE. The third, registered in April 2018, by Angélica Valencia is limited to preterm neonates and is evaluating the type of method used for the recognition of these early movements and not the relationship of GM Assessments in the diagnosis of NE to neuromotor outcomes^15^. Thus, a gap exists in the literature to clearly identify the evidence for our population.

The objective of this review is to evaluate the effectiveness of the General Movements Assessment versus traditional neurological examination on neuromotor outcomes, namely cerebral palsy, in term and late-preterm newborns with a diagnosis of NE.

# Review Question

The question of this scoping review is: What is the predictive value of the GM Assessment for the diagnosis of cerebral palsy at two years of age in neonates born at term or late-preterm with a diagnosis of Neonatal Encephalopathy?

# Keywords

neonatal encephalopathy; general movement; Prechtl; hypoxia-ischemia;  cerebral palsy.

# Inclusion Criteria

### Participants

The review will consider studies that include neonates ≥ 34+0 weeks GA diagnosed with neonatal encephalopathy with a GM Assessment done between birth to 6 months of life. Studies in the English language only will be considered as there is no team member with adequate language skills to translate from any other language.

Reviews with neonates born at < 34 weeks GA, those without a diagnosis of NE and those born with life threatening congenital abnormalities, congenital viral infections, an abnormal karyotype and metabolic disorders will be excluded. Those studies without a GM Assessment will also be excluded.

### Concept

GM Assessment as a predictor of cerebral palsy at 2 years of age.

### Context

This review will consider studies that reported on newborns with an existing diagnosis of Neonatal Encephalopathy managed in hospitals and diagnosed by the standard of care assessment of a neurological history and examination. Studies will be considered from all countries that have outcomes reported in the acute neonatal and in the follow-up period at 2 years of age.

### Types of Sources

This scoping review will consider both experimental and quasi-experimental study designs including randomized controlled trials, non-randomized controlled trials, before and after studies and interrupted time-series studies. In addition, analytical observational studies including prospective and retrospective cohort studies, case-control studies and analytical cross-sectional studies will be considered for inclusion. This review will also consider descriptive observational study designs including case series, individual case reports and descriptive cross-sectional studies for inclusion.

Qualitative studies will also be considered that focus on qualitative data including, but not limited to, designs such as phenomenology, grounded theory, ethnography, qualitative description, action research and feminist research.

In addition, systematic reviews that meet the inclusion criteria will also be considered.

Text and opinion papers will not be considered for inclusion in this scoping review as this is a highly specific and medical topic.

Studies published in English will be included. Studies published from at least 1970 will be included as this is around the time when the GM Assessments were first introduced in neonatology as a potential predictor of neuromotor outcomes^16^.

# Methods

The proposed systematic review will be conducted in accordance with the Joanna Briggs Institute methodology for scoping reviews^17^.

### Search strategy

The search strategy will aim to locate both published and unpublished studies. An initial limited search of MEDLINE, Embase and PsychINFO was undertaken to identify articles on the topic. The text words contained in the titles and abstracts of relevant articles, and the index terms used to describe the articles were used to develop a full search strategy for  Pubmed and CINAHL (see Appendix 1). The search strategy, including all identified keywords and index terms, will be adapted for each included information source. The reference list of all studies selected for critical appraisal will be screened for additional studies.

### Information sources

The databases to be searched include CINAHL and Pre-CINAHL, Cochrane, EMBASE, Joanna Briggs Institute EBP, Ovid MEDLINE,  PsycINFO and PubMed. Sources of unpublished studies and gray literature will not be searched as described above.

### Study selection

Following the search, all identified citations will be collated and uploaded into EndNote basic and duplicates removed. Titles and abstracts will then be screened by two independent reviewers for assessment against the inclusion criteria for the review. Potentially relevant studies will be retrieved in full and their citation details imported into the Joanna Briggs Institute System for the Unified Management, Assessment and Review of Information (JBI SUMARI) (Joanna Briggs Institute, Adelaide, Australia)^17^. The full text of selected citations will be assessed in detail against the inclusion criteria by two independent reviewers. Reasons for exclusion of full text studies that do not meet the inclusion criteria will be recorded and reported in the systematic review. Any disagreements that arise between the reviewers at each stage of the study selection process will be resolved through discussion, or with a third reviewer. The results of the search will be reported in full in the final systematic review and presented in a Preferred Reporting Items for Systematic Reviews and Meta-analyses (PRISMA) flow diagram^18^.

### Data Extraction

Data will be extracted from papers included in the scoping review by two independent reviewers using a data extraction tool developed by the reviewers. The data extracted will include specific details about the population, concept, context, study methods and key findings relevant to the review objective. A draft charting table is provided (see Appendix 2). The draft data extraction tool will be modified and revised as necessary during the process of extracting data from each included study. Modifications will be detailed in the full scoping review report. Any disagreements that arise between the reviewers will be resolved through discussion, or with a third reviewer. Authors of papers will be contacted to request missing or additional data, where required. 

### Data Presentation

The extracted data will be presented in diagrammatic or tabular form in a manner that aligns with the objective of this scoping review. A narrative summary will accompany the tabulated and/or charted results and will describe how the results relate to the reviews objective and question/s.

# Acknowledgements

This review will contribute to a Master in Healthcare Quality degree for JS.

# Funding

There is no funding required for this review.

# Conflicts of interest

There is no conflict of interest in this project.

# References

1. Health Quality Ontario’s System, Quality Advisory Committee. Quality matters: realizing excellent care for all. Queen’s Printer for Ontario; 2017.

2. Glass HC. Hypoxic-ischemic encephalopathy and other neonatal encephalopathies. [Review]. Continuum (Minneap Minn). 2018 Feb;57–71.

3. American Academy of Pediatrics, Committee on Fetus and Newborn. Hypothermia and neonatal encephalopathy. Pediatrics. 2014 Jun;133(6):1146–50.

4. Staub K, Baardsnes J, Hébert N, Hébert M, Newell S, Pearce R. Our child is not just a gestational age. A first‐hand account of what parents want and need to know before premature birth. Acta Paediatr. 2014;103(10):1035–38.

5. Banihani R TCP. Neonatal Encephalopathy. In: Needelman H JB, editor. Follow-Up for NICU Graduates. 2018. p. 155–78.

6. Bosanquet M, Copeland L, Ware R, Boyd R. A systematic review of tests to predict cerebral palsy in young children. Dev Med Child Neurol. 2013;55:418–26.

7. Hadders-Algra M. General Movements: A Window for early identification of children at high risk for developmental disorders. J Pediatr. 2004 May 11;145(2 Supplement):S12–8.

8. Kwong AKL, Fitzgerald TL, Doyle LW, Cheong JL, Spittle AJ. Predictive validity of spontaneous early infant movement for later cerebral palsy: a systematic review. Dev Med Child Neurol. 2018 Feb 22;60(5):480-489.

9. Burger M, Louw QA. The predictive validity of general movements – a systematic review. Eur J Paediatr Neurol. 2009;13(5):408–20.

10. Spittle AJ, Doyle LW, Boyd RN. A systematic review of the clinimetric properties of neuromotor assessments for preterm infants during the first year of life. Dev Med Child Neurol. 2008 Apr 8;50(4):254–66.

11. Darsaklis V, Snider LM, Majnemer A, Mazer B. Predictive validity of Prechtl’s method on the qualitative assessment of general movements: a systematic review of the evidence. Dev Med Child Neurol. 2011 Jun 17;53(10):896–906.

12. Hadders-Algra M. Evaluation of motor function in young infants by means of the assessment of general movements: a review. Pediatr Phys Ther. 2001 Apr 1;13(1):27–36.

13. Kwong AKL, Fitzgerald TL, Spittle AJ, Cheong JL, Doyle LW, Einspiela C. A systematic review of the predictive validity of observational early infant motor assessments for subsequent cerebral palsy. PROSPERO International prospective register of systematic reviews. 2016; Available from: http://www.crd.york.ac.uk/PROSPERO/display_record.php?ID=CRD42016042551.

14. Raghuram K, Orlandi S, Church P, Chau T, Uleryk E, Pechlivanoglou P, Shah V. Can an automated general movements assessment be used to predict motor impairment in high-risk infants? A systematic review and meta-analysis of diagnostic accuracy. PROSPERO International prospective register of systematic reviews. 2018 Apr 16; Available from: <http://www.crd.york.ac.uk/PROSPERO/display_record.php?ID=CRD42018087892>.

15. Valencia A. Discriminative and predictive validity of the general movements assessment: a systematic review. PROSPERO International prospective register of systematic reviews. 2018 Feb 14;Available from: <http://www.crd.york.ac.uk/PROSPERO/display_record.php?ID=CRD42018088724>.

16. Einspieler C, Prechtl HFR. Prechtl’s Method on the Qualitative Assessment of General Movements in Preterm, Term and Young Infants. 1st ed. Mac Keith Press: British Library Cataloguing-in-Publication data; 2004.

17. The Joanna Briggs Institute. The System for the Unified Management, Assessment and Review of Information (SUMARI) [Internet]. 2017 [cited 2019]. Available from: <https://www.jbisumari.org/>

18. Moher D, Liberati A, Tetzlaff J, Altman DG. Preferred Reporting Items for Systematic Reviews and Meta-Analyses: The PRISMA Statement. [Internet]. 2009 [cited 2019]. Available from: <http://prisma-statement.org/PRISMAStatement/FlowDiagram.aspx>

# Appendices

### Appendix I: Search strategy

Search conducted on Ovid MEDLINE(R), Ovid MEDLINE(R) Daily and Epub Ahead of Print, In-Process & Other Non-Indexed Citations 1946 to Present.

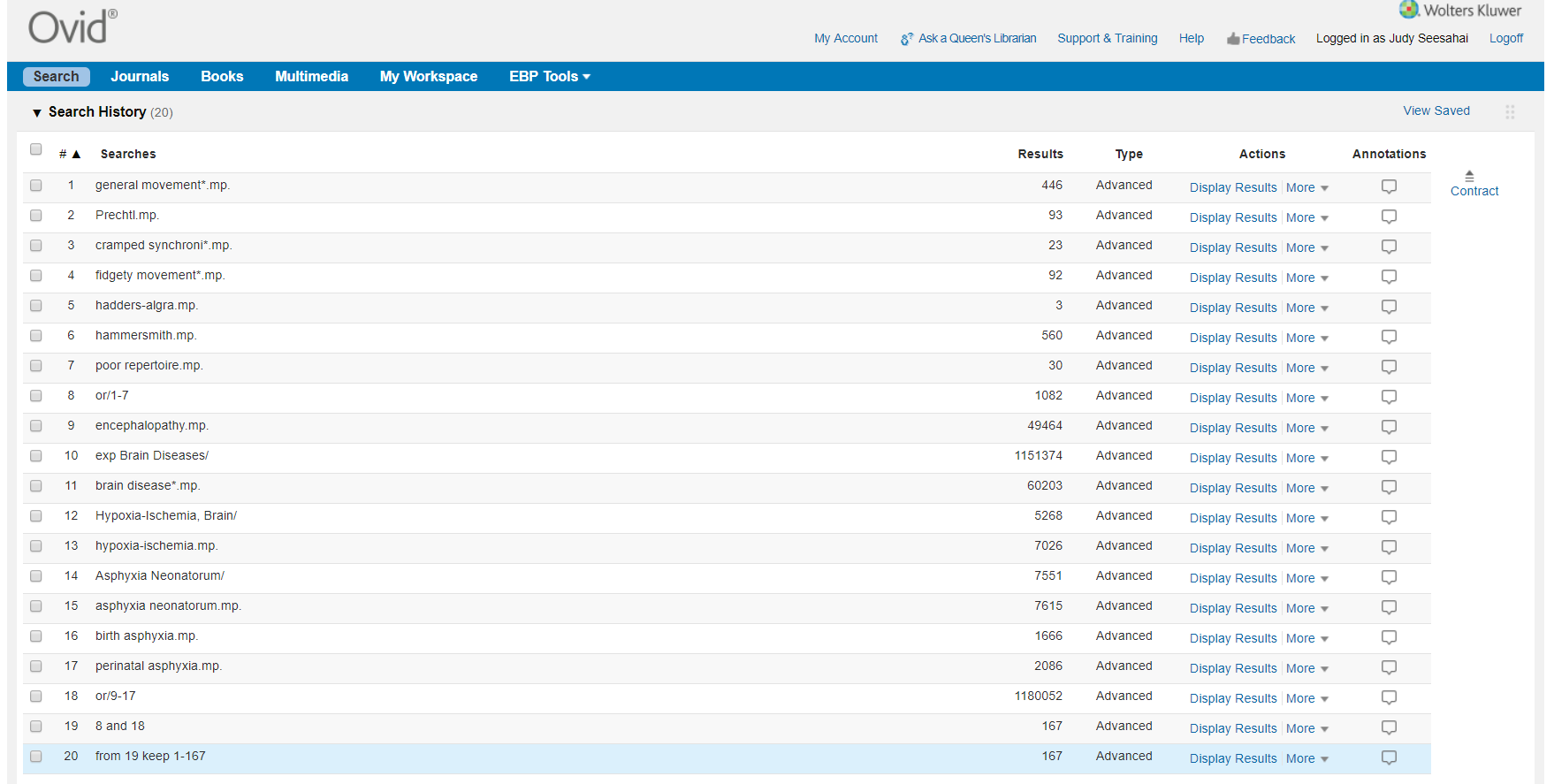


Search conducted on May 22nd, 2019.

### Appendix II: Data extraction instrument

The standardized tool from the JBI SUMARI will be used for data extraction^17^.
