# Supplementary material for: The general movements assessment in term and late-preterm infants diagnosed with neonatal encephalopathy, as a predictive tool of cerebral palsy by 2 years of age: a scoping review protocol
Source: Syst Rev. 2020 Jul 4;9:154. doi: 10.1186/s13643-020-01358-x (PMC7335433; doi:10.1186/s13643-020-01358-x)
Supplement: Supplementary file 2 — Additional file 2. Ovid MEDLINE search. Search conducted on Ovid MEDLINE(R), Ovid MEDLINE(R) Daily and Epub Ahead of Print, In-Process & Other Non-Indexed Citations 1946 to Present [file 13643_2020_1358_MOESM2_ESM.docx]

Additional file 2: Ovid MEDLINE search

Search conducted on Ovid MEDLINE(R), Ovid MEDLINE(R) Daily and Epub Ahead of Print, In-Process & Other Non-Indexed Citations 1946 to Present.

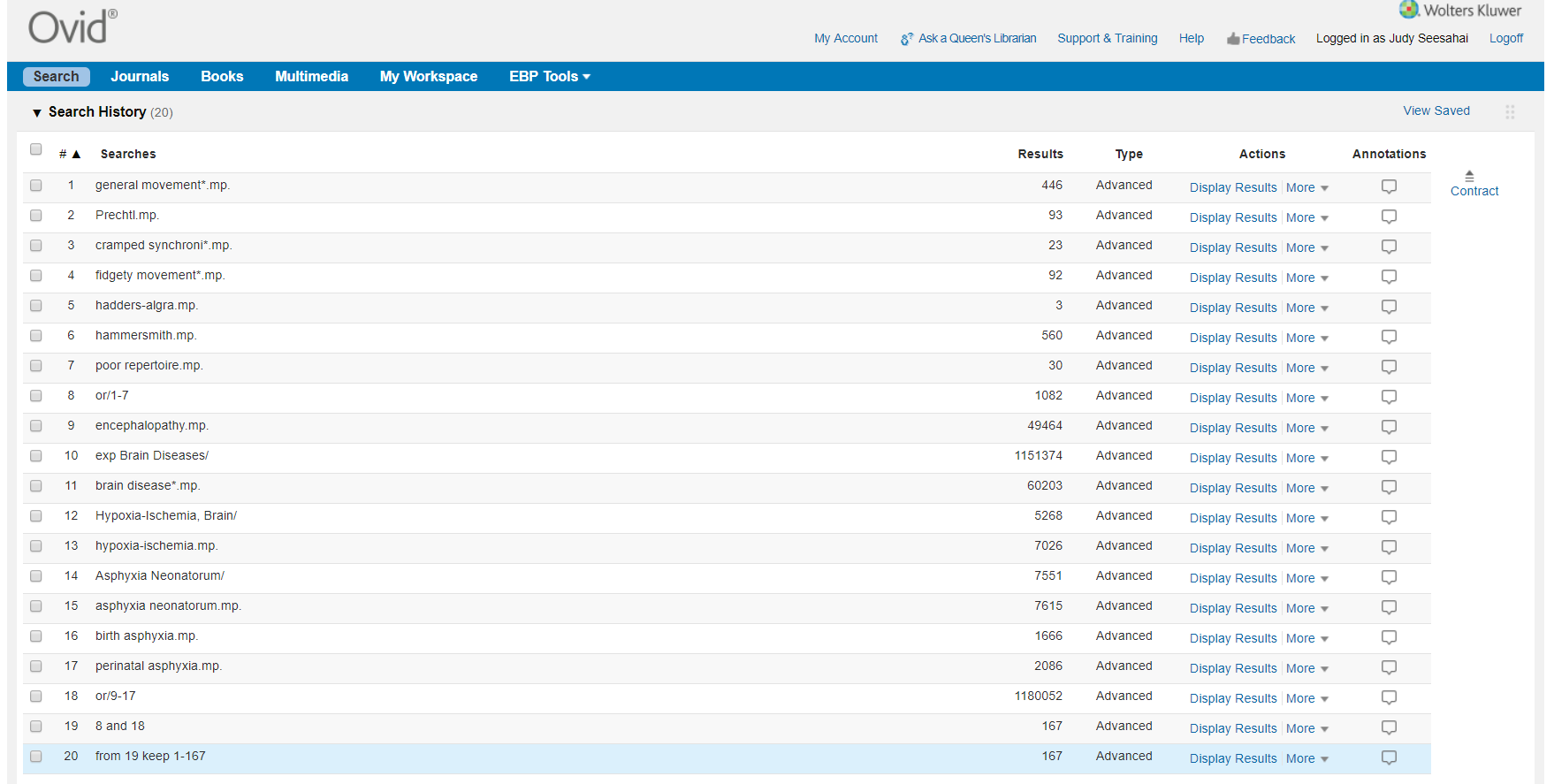


Search conducted on May 22nd, 2019.
